# Cardiovascular Parameters in a Swine Model of Normobaric Hypoxia Treated With 5-Hydroxymethyl-2-Furfural (5-HMF)

**DOI:** 10.3389/fphys.2019.00395

**Published:** 2019-04-18

**Authors:** Richard Thomas Mahon, Geoffrey E. Ciarlone, Nicholas G. Roney, Joshua M. Swift

**Affiliations:** Department of Undersea Medicine, Naval Medical Research Center, Silver Spring, MD, United States

**Keywords:** oxyhemoglobin affinity, P_50_, oxygen, cardiopulmonary, arterial pressure

## Abstract

**Introduction:**

The consequences of low partial pressure of O_2_ include low arterial O_2_ saturations (SaO_2_), low blood O_2_ content (CaO_2_), elevated mean pulmonary artery pressure (PAP), and decreased O_2_ consumption VO_2_. 5-hydroxymethyl-2-furfural (5-HMF) binds to the N-terminal valine of hemoglobin (HgB) and increases its affinity to O_2_. We used an instrumented, sedated swine model to study the effect of 5-HMF on cardiovascular parameters during exposure to acute normobaric hypoxia (NH).

**Methods:**

Twenty-three sedated and instrumented swine were randomly assigned to one of three treatment groups and received equal volume of normal saline (VEH), 20 mg/kg 5-HMF (5-HMF-20) or 40 mg/kg 5-HMF (5-HMF-40). Animals then breathed 10% FiO_2_ for 120 min. Parameters recorded were Cardiac Output (CO), Mean Arterial Blood Pressure (MAP), Heart Rate (HR), Mean Pulmonary Artery Pressure (PAP), SaO_2_ and saturation of mixed venous blood (SvO_2_). The P_50_ was measured at fixed time intervals prior to and during NH.

**Results:**

5-HMF decreased P_50_. In the first 30 min of NH, treatment with 5-HMF-20 and 5-HMF-40 resulted in a (1) significantly smaller decrement in SaO_2_ and SvO_2_, (2) significantly lower HR and CO, and (3) smaller increase in PAP compared to VEH. In the 120 min of NH there was a trend toward improved mortality with 5-HMF treatment.

**Conclusion:**

5-HMF treatment decreased P_50_, improved SaO_2_, and mitigated increases in PAP in this swine model of NH.

## Introduction

The reduction of barometric pressure at altitude is associated with reduced partial pressure of ambient Oxygen (PO_2_). With lower ambient PO_2_, it can be anticipated that alveolar (PAO_2_), arterial O_2_ (PaO_2_) and blood O_2_ content (CaO_2_) will decrease accordingly, resulting in a widely recognized decrease in maximal O_2_ consumption (VO_2_) (Dill and Adams, 1972; Lucas et al., 2011). In humans, with acute exposure to altitude, maximal VO_2_ decrements are measurable at elevations even as low as 580 m (Gore et al., 1997) and are decreased by 25% at about 5000 m (Calbet et al., 2003). Such decrements also appear to extend to native low altitude animals exposed to hypoxia with swine showing a 30% decrement in VO2 max while breathing an FiO2 of 0.125 (Hopkins et al., 2007) and similar decrements noted in native low altitude rodent exposed to altitude (Gonzalez et al., 1998).

In acute altitude exposure, some decrease in exercise capacity can be simply attributed to a decrease in CaO_2_ (Gore et al., 1997). Though acclimation to high altitude is highly variable (Keys et al., 1938; Weil et al., 1970; Friedman et al., 2005); with the general response of increased hemoglobin (HgB) and improved arterial O_2_ saturation (SaO2) (from increased minute ventilation), CaO_2_ may approach sea level values (Grocott et al., 2009). Yet maximal aerobic exercise and VO_2_ remains decreased (Cerretelli, 1976; Calbet et al., 2003). Cardiovascular limitations include decrease in cardiac output (CO), heart rate (HR) and right ventricular dysfunction from hypoxic pulmonary vasoconstriction (Bärtsch and Gibbs, 2007; Martin et al., 2015). Other limits to increased exercise capacity at altitude include a lung O_2_ diffusion limit and/or a blood to muscle diffusion O_2_ limit (Dávila-Román et al., 1997; Calbet and Lundy, 2009). The invocation of such mechanisms requires that the role of HgB in O_2_ transport must be considered (Stray-Gundersen et al., 2001).

Oxygen’s affinity for binding to hemoglobin is reflected in the O_2_-HgB (O-H) dissociation curve and expressed as the partial pressure of O_2_ at which HgB is 50% saturated (P_50_). Leftward shifts of the O-H curve (lower P_50_) favor “on loading” of O_2_ to HgB at the alveoli, but theoretically decreases the ability of O_2_ “off-loading” at the tissue level, whereas a rightward shift (higher P_50_) decreases “loading” of O_2_ at the alveolar level, but potentially improves “off-loading” at the tissue level (Stray-Gundersen et al., 2001).

HgB affinity for oxygen is modified during acute altitude exposure (Storz and Moriyama, 2008). At altitude, a decreased partial pressure of carbon dioxide (pCO_2_) (by increased ventilation) and corresponding increase in pH (less H+ ions) should affect a shift of the O-H dissociation curve to the left (Luks, 2015). However, the well-defined increase in 2,3-diphosphoglycerate (2,3 DPG) with acclimation favors a right shift of the O-H curve (Collins et al., 2015). In humans, whether any change in P_50_ is adaptive, maladaptive, or simply a consequence of hypobaric hypoxia (HH), is unknown.

In contrast to limited human studies, left shifts in O-H curve are well described in avian species that have genetically adapted to high altitude (Stray-Gundersen et al., 2001). For example, the bar-headed goose which must traverse over the Himalayan mountains has a lower P_50_ (left shift of O-H dissociation curve) when compared to its low altitude relatives (Macdonald, 1977). Although such adaptations are less evident in mammals, pharmacologically decreasing the P_50_ has led to improved tolerance to severe hypoxia in certain species (Scott, 2011).

### 5-Hydroxymethyl-2-Furfural (5-HMF)

5-HMF reduces P_50_ via allosteric modification of HgB by forming a high affinity Shiff-base HgB adduct with the N-terminal alpha valine of the HgB molecule (Yalcin and Cabrales, 2012). In hamsters, 5-HMF led to a decrease in P_50_ that was associated with improved arterial O_2_ saturation (SaO_2_), CO and oxygen delivery (DO_2_), compared to controls during exposure to 0.10 and 0.05 fraction of inspired Oxygen (FiO_2_) (Scott, 2011).

Given the seeming benefits of increased O-H affinity in adapted high altitude animals we studied the acute cardiopulmonary effects of 5-HMF in a large animal (swine) model of normobaric hypoxia (NH) exposed to an FiO_2_ of 0.10. We hypothesized that an acute increased O-H affinity would improve SaO_2,_ mixed venous O_2_ (SvO_2_) and mean pulmonary artery pressure (PAP).

## Materials and Methods

### Ethics Statement

All experiments were conducted according to the principles set forth in the “Guide for the Care and Use of Laboratory Animals,” Institute of Laboratory Animal Resources, National Research Council, National Academy Press, 2011. The study protocol was reviewed and approved by the Walter Reed Army Institute of Research/Naval Medical Research Center Institutional Animal Care and Use Committee in compliance with all Federal regulations governing the protection of animals in research. The health status of animals was monitored daily and the research was conducted in a facility accredited by the Association for Assessment and Accreditation of Laboratory Animal Care-International (AAALACI). Euthanasia was carried out in accordance with the recommendations and guidelines of the American Veterinary Medical Association.

### Animals and Experimental Design

Castrated Yorkshire swine (*N* = 23) were procured from a single vendor for all experiments (Animal Biotech Industries, Danboro, PA, United States) and acclimatized for at least 5 days prior to any experiment. Free access to water and food was allowed (“Lab Diet,” from PMI Nutrition LLC, Brentwood, MO, United States) while undergoing a daily on/off light schedule of 12 h per 24 h day.

### Animal Surgical Preparation

Animals were transported from their holding pens to a surgical suite and anesthetized initially with Ketamine/Xylazine [(5–20/0.5–2 mg/kg) (Ketamine Putney Inc, Portland, ME, United States (Xylazine - Anased Lloyd Pharmaceuticals, Shenandoah, IA, United States))] by intramuscular injection. A 23-g, 1-inch ear-vein catheter was placed (BD Angiocath, Beckton Dickinson, Infusion Therapy Systems, Sandy, UT, United States). Afterward the animal was maintained on isoflurane inhalant (2–5%) via endotracheal tube, maintained in a supine position and body temperature maintained at 100–102°F. FiO_2_ was maintained at 0.21 unless clinically warranted.

**FIGURE 1:**
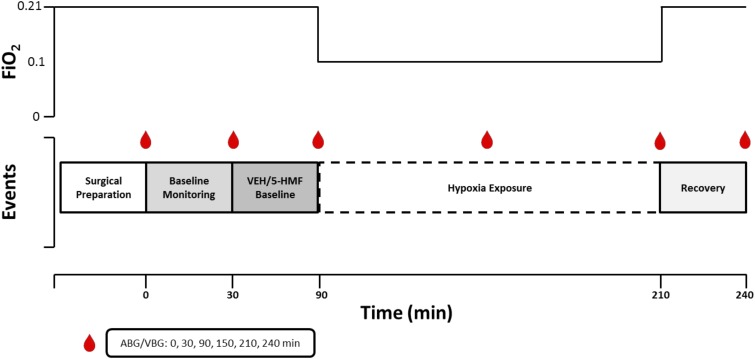
Graphical representation of experimental design. The experiment consisted of a surgical epoch and four subsequent experimental periods. Baseline Monitoring (30 min of baseline monitoring; FiO_2_ = 0.21); Drug Baseline [60 min of baseline monitoring after VEH or 5-HMF (20 or 40 mg/kg) administration; FiO_2_ = 0.21]; Hypoxia Exposure (120 min of severe hypoxia exposure while sedated; FiO_2_ = 0.10); Recovery (30 min of recovery post-hypoxia; FiO_2_ = 0.21). ABG, arterial blood gas; VBG, venous blood gas; FiO_2_, Fraction of Inspired Oxygen.

### Electrocardiogram and Pulmonary Arterial Catheter Placement

The animal was fitted with electrocardiogram leads (ECG) as well as pulse oximetry to measure SaO_2_ (Smiths Medical PM, Waukesha, WI, United States). After an acceptable plane of anesthesia was obtained, the animal underwent placement of pulmonary artery catheter (PAC) and arterial lines. Using known landmarks, an 18-gauge needle was inserted into the internal jugular vein and using a modified Seldinger technique the vessel was dilated over a guide wired and a 9 Fr introducer Sheath was inserted (StarFlex InSitu Technologies, St. Paul, MN, United States). In animals where the landmark technique was not successful, a cut-down technique was used beginning with a 5–7 cm craniocaudal incision made over the right internal jugular furrow. Then, blunt and sharp dissection isolated the jugular vein and subsequently the 9-Fr introducer sheath was inserted. The wound was then closed with staples. Next, the PAC, with continuous cardia output (Swan-Ganz CCOmboV, Edwards Life Sciences, Irvine, CA, United States), was inserted and placed into the pulmonary artery (using standard waveforms), and a wedge position with balloon inflation was confirmed. Continuous CO was confirmed, mixed venous O_2_ saturation (SvO_2_) was calibrated, and pressure measurements were tared to zero.

### Left and Right Femoral Artery Access for Blood and Arterial Pressure Analyses

Systemic arterial access was achieved via a superficial branch of the left femoral artery medial and proximal to the knee. A small, longitudinal incision was made and the arterial branch gently isolated using blunt and sharp dissection. A 14 g catheter (Arrow International) was then placed into the artery and secured with silk ligatures. For continuous, uninterrupted MAP recordings, an intravascular solid-state transducer (Mikro-Cath^TM^, Millar, Houston, TX, United States) was placed in the right superficial branch of the femoral artery and sutured into place. Next, a local injection of bupivacaine (5–10 cc with a 23 g needle) was infiltrated around the surgical sites. Buprenorphine 0.1 mg/kg was given IM, isoflurane was discontinued, and the animal was transferred to a Panepinto sling. The animal was then infused with diazepam 10–20 mg/hour intravenously through the duration of the experiment. Once the animal was breathing spontaneously and demonstrated ability to protect airway, tracheal extubation occurred.

### Experimental Procedures

A graphical depiction of the experiment is shown in Figure 1. Animals were randomized on the day of experiment to one of three groups; VEH: control group given 100 ml of Normal Saline (NS) intravenously; 5-HMF-20: 20 mg/kg 5-HMF given intravenously and 5-HMF-40: 40 mg/kg 5-HMF given intravenously. 5-hydroxymethyl-2-furfural (5-HMF; W501808; Sigma-Aldrich, St. Louis, MO, United States) was prepared under sterile conditions by dissolving the desired quantity in 100 mL normal saline (NS; 0.9% NaCl) and passed through a 0.22-micron filter. Fresh solution was prepared prior to each experiment.

After achieving a steady and acceptable plane of sedation and breathing room air, the animal entered into a 30-min baseline monitoring phase. Subsequently, the animal was administered 5-HMF or NS based on random group and again was monitored while breathing room air for 60 min. After this period, the animal breathed 10% O_2_ via a modified veterinary anesthesia mask which included an external rubber skirt that ensured minimal gas leak. Specifically, using a Hans Rudolph three-way valve (Hans Rudolph, Inc., Shawnee, KS, United States) connected to a 20 L Douglas bag continually filled with 10% O_2_ at a flow of 40 L per minute. The 10% O_2_ mixture was achieved by mixing air and nitrogen with continuous in-line monitoring (Alpha Omega Series 9600, Alpha Omega Instruments, Lincoln, RI, United States) prior to reaching the gas inlet port of the Douglas bag. The animal was then exposed to NH for a 120-min epoch. After NH exposure, animals returned to room air through removal of the mask and were monitored for 30 min during recovery. At the end of the experiment, the animal was humanely euthanized with euthanasia solution (Euthasol - Virbac AH, Fort Worth, TX, United States).

During the course of the experiment, animals received as needed, bolus doses of 5–10 mg diazepam beyond the previously noted diazepam infusion to maintain comfort. Cardiac Output (CO), Mean Arterial Blood Pressure (MAP), Heart Rate (HR), Mean Pulmonary Artery Pressure (PAP), SaO_2_, and SvO_2_ were continuously displayed to research staff and manually recorded every 5 min. Arterial blood gas and mixed venous blood gas were measured on an automated blood gas machine (ABL800 FLEX, Radiometer America, Brea, CA, United States) and were recorded at 0, 30, 90,150, 210, and 240 min during the experiment.

As previously described (Luks, 2015), O-H curve and P_50_ were measured by deoxygenation of O_2_-equilibrated samples in Hemox buffer at 37°C using a Hemox Analyzer (TCS Scientific Corporation, New Hope, PA, United States). Hemox buffer pH was adjusted to match the arterial pH with Tris and BisTris Buffers. The P_50_ was determined at 30, 90, 150, 210, and 240 min of the experimental protocol.

### Statistical Analyses

All data are presented as mean ± SE (figures) or SD (tables), and their statistical relationships were evaluated using the statistical package GraphPad Prism (v.7; La Jolla, CA, United States). A one-factor ANOVA was used to assess (1) the effect of each dose of 5-HMF at each time point vs. VEH and (2) the effects of 5-HMF ± hypoxia over time compared to baseline values. Due to the collection of blood for CBC analysis at only two time points following baseline, an unpaired *t*-test was used to assess the effects of 5-HMF ± hypoxia over time compared to baseline. When a significant interaction was observed, pairwise comparisons were performed using Tukey’s (dose) or Dunnett’s (time) multiple comparisons test. Statistical significance was defined as *p* ≤ 0.05 prior to statistical analysis.

**FIGURE 2:**
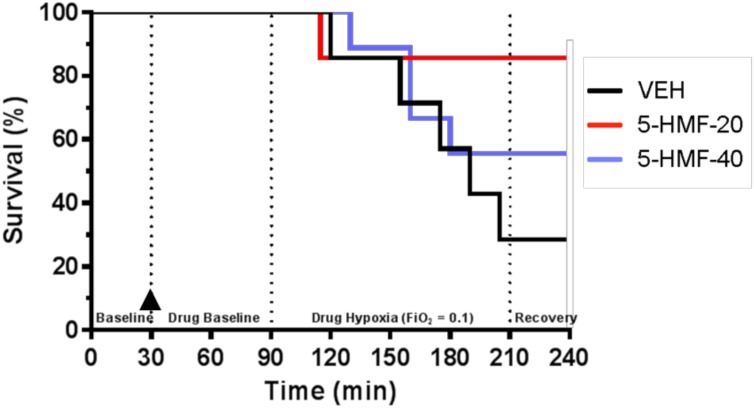
Animal survival during experimental protocol. Groups: VEH (100 ml NS; *n* = 7), 5-HMF-20 (20 mg/kg 5-HMF dissolved in 100 ml NS; *n* = 7) and 5-HMF-40 (40 mg/kg 5HMF dissolved in 100 ml NS; *n* = 9). 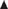 denotes time (30 min) of VEH or 5-HMF administration. There was no significant effect of 5-HMF on animal survival during the experiment (*p* = 0.15).

## Results

### Effects of 5-HMF Treatment on Survival During Hypoxia

Twenty-three swine began the investigation and were randomly assigned to one of three treatment groups: VEH (*n* = 7; 32.2 ± 1.6 kg), 5-HMF-20 (*n* = 7; 31.0 ± 2.7 kg), and 5-HMF-40 (*n* = 9; 33.8 ± 2.9 kg). There was no significant difference in body mass between any of the treatment groups. For the 120 min NH exposure, survival was 29% in VEH, 86% in 5-HMF-20 and 56% in 5-HMF-40 treated animals (Figure 2). Those animals that survived the entire hypoxia phase, also tolerated the recovery phase well.

**Table 1:** Effect of 5-HMF on oxyhemoglobin affinity (P_50_) during normoxia and hypoxia exposures in swine.

Treatment	Time (FiO_2_)				
	30 (0.21)	90 (0.21)	150 (0.10)	210 (0.10)	240 (0.21)
VEH	43.05 ± 2.03	39.48 ± 4.85	42.00 ± 4.02	39.47 ± 3.44	40.97 ± 4.83
5-HMF-20	43.48 ± 3.23	42.43 ± 1.31	38.08 ± 8.26	23.47 ± 5.52^#†^	22.18 ± 9.17^#†^
5-HMF-40	41.10 ± 3.23	36.20 ± 8.14	33.79 ± 9.02^#‡^	28.47 ± 11.76^#†^	24.23 ± 12.16^#†^

*Values in mmHg. Groups: VEH (100 ml NS), 5-HMF-20 (20 mg/kg 5HMF dissolved in 100 ml NS) and 5-HMF-40 (40mg/kg 5HMF dissolved in 100 ml NS). FiO_2_, Fraction of Inspired Oxygen. All values are mmHg mean ± SD. ^#^ vs. VEH (same time point; p ≤ 0.01); ^‡^ vs. Baseline (30 min measurement; p ≤ 0.05); vs. ^†^ Baseline (p ≤ 0.01).*

### 5-HMF Significantly Decreases P_50_

Baseline P_50_ was 43.05 mmHg (±2.03), 43.48 (±3.23), and 41.10 (±3.23) in VEH, 5-HMF-20 and 5-HMF-40 groups respectively and not significantly different. VEH treatment did not significantly affect arterial blood P_50_ during normoxia or NH exposures (Table 1). The P_50_ of the 5-HMF-20 group was significantly decreased when compared to baseline and VEH starting at 180 min after infusion with the lowest P_50_ noted at the end of the experiment 22.18 mmHg (±9.17). Similarly, the P_50_ of the 5-HMF-40 showed a significant decrease when compared to baseline and VEH starting at 120 min after administration 33.79 mmHg (±9.02) that was observed through the entire experiment with a final P_50_ of 24.23 mmHg (±12.16).

**FIGURE 3:**
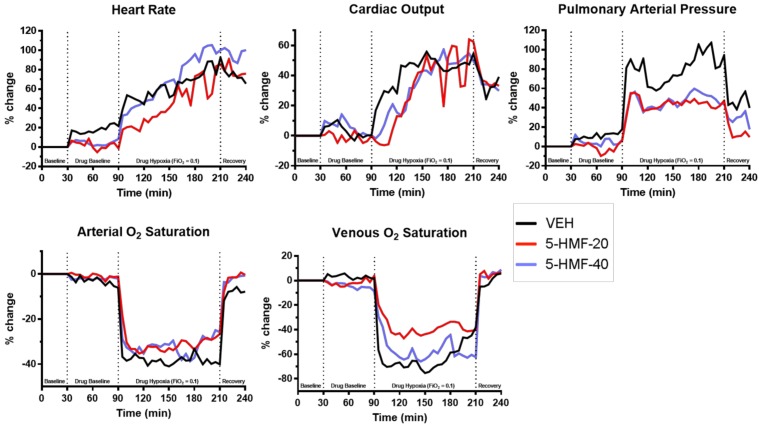
Cardiopulmonary outcomes during normoxia and hypoxia exposure in swine. Groups: VEH (100 ml NS), 5-HMF-20 (20 mg/kg 5-HMF dissolved in 100 ml NS) and 5-HMF-40 (40 mg/kg 5-HMF dissolved in 100 ml NS). All values are mean %-change in parameter (vs. mean baseline value) recorded every 5 min during each phase of the experiment.

**Table 2:** Cardiopulmonary outcomes during normoxia and hypoxia exposures in swine.

Treatment	Time (FiO_2_)							
	0–30 (0.21)	35–60 (0.21)	65–90 (0.21)	95–120 (0.10)	125–150 (0.10)	155–180 (0.10)	185–210 (0.10)	215–240 (0.21)
*Heart Rate (bpm)*		*% change from baseline*						
VEH	104.54 ± 9.72	15.28 ± 8.15	20.95 ± 9.84	47.57 ± 16.98^‡^	57.84 ± 16.08^‡^	58.26 ± 38.74^‡^	73.99 ± 34.55^‡^	73.01 ± 29.90^‡^
5-HMF-20	100.67 ± 15.99	4.12 ± 15.74	-0.18 ± 13.08^#^	20.58 ± 11.46^∗^	33.54 ± 16.08^#^	60.93 ± 32.27^‡^	81.02 ± 35.75^‡^	77.91 ± 43.94^‡^
5-HMF-40	107.21 ± 26.76	4.96 ± 9.66^#^	4.06 ± 16.31^∗^	39.71 ± 26.10^‡^	53.60 ± 37.51^‡^	72.27 ± 29.71^‡^	99.60 ± 17.26^‡^	96.09 ± 13.16^‡^
*Cardiac Output (L/min)*		*% change from baseline*						
VEH	6.07 ± 1.09	6.79 ± 11.98	−0.52 ± 14.74	26.56 ± 27.16	47.59 ± 27.72^‡^	48.38 ± 20.05^‡^	45.89 ± 26.09^‡^	34.99 ± 4.87
5-HMF-20	4.76 ± 1.09	−0.64 ± 14.97	−1.29 ± 18.92	1.07 ± 16.83^#^	29.89 ± 22.59	48.54 ± 31.15^‡^	63.86 ± 42.80^‡^	36.97 ± 21.59^‡^
5-HMF-40	5.74 ± 1.15	9.34 ± 18.39	2.11 ± 22.37	9.68 ± 18.14^#^	24.94 ± 23.30	42.55 ± 22.25^‡^	50.47 ± 31.91^‡^	34.92 ± 28.21^‡^
*Stroke Volume (mL)*		*% change from baseline*						
VEH	58.11 ± 9.69	−6.79 ± 14.06	−17.42 ± 12.69	−13.70 ± 15.85	−5.68 ± 15.48	−8.33 ± 23.29	−12.82 ± 21.97	−20.55 ± 12.28
5-HMF-20	47.67 ± 10.3	−2.92 ± 19.81	0.01 ± 16.37	−16.44 ± 15.82	−0.71 ± 19.86	−6.76 ± 11.83	−10.89 ± 14.05	−21.44 ± 16.36
5-HMF-40	56.08 ± 15.73	4.02 ± 15.35	−1.80 ± 17.58	−20.59 ± 14.18^‡^	−16.32 ± 17.73^‡^	−16.18 ± 11.28^‡^	−24.99 ± 15.08^‡^	−30.33 ± 19.17^‡^
*Pulmonary Artery Pressure (mmHg)*		*% change from baseline*						
VEH	21 ± 5.25	9.49 ± 15.62	13.07 ± 17.53	80.37 ± 29.42^‡^	64.29 ± 30.03^‡^	77.15 ± 51.13^‡^	91.39 ± 47.33^‡^	45.52 ± 36.50
5-HMF-20	24.41 ± 7.24	1.30 ± 14.92	−2.81 ± 18.83	45.07 ± 28.13^#‡^	42.66 ± 26.26^‡^	45.86 ± 28.94^‡^	43.33 ± 25.54^∗‡^	13.28 ± 26.93
5-HMF-40	24 ± 5	5.07 ± 10.13	4.50 ± 18.12	44.16 ± 21.04^#‡^	41.03 ± 16.37^‡^	46.22 ± 21.03^‡^	49.35 ± 15.35^‡^	28.25 ± 19.96^‡^
*Mean Arterial Pressure (mmHg)*		*% change from baseline*						
VEH	95.96 ± 14.69	10.21 ± 10.67	11.75 ± 15.24	19.95 ± 33.30	16.01 ± 25.39	1.01 ± 17.03	−2.64 ± 41.69	−12.33 ± 0.00
5-HMF-20	110.43 ± 24.23	4.13 ± 11.36	5.72 ± 14.86	−4.16 ± 19.42	0.57 ± 20.15	−2.22 ± 24.76	−6.78 ± 33.65	−17.87 ± 15.62
5-HMF-40	103.79 ± 9.27	5.46 ± 4.50	4.34 ± 3.29	1.13 ± 18.55	0.14 ± 33.85	−10.12 ± 37.65	−1.12 ± 6.74	−17.21 ± 1.34
*Arterial O_2_ Saturation (%)*		*% change from baseline*						
VEH	95.68 ± 3.17	−2.37 ± 3.55	−4.06 ± 5.16	−39.13 ± 2.50^‡^	−39.5 ± 8.66‡	−38.40 ± 6.20‡	−40.04 ± 7.67‡	−8.15 ± 10.07
5-HMF-20	96.08 ± 1.91	−0.49 ± 1.56	−1.34 ± 1.51	−30.77 ± 3.92^∗‡^	−33.01 ± 7.2‡	−31.74 ± 8.83‡	−28.8 ± 6.01^∗^‡	−2.03 ± 2.25
5-HMF-40	96.06 ± 1.94	−2.21 ± 2.01	−1.91 ± 2.69	−33.11 ± 2.62^∗‡^	−31.88 ± 9.86‡	−33.31 ± 7.72‡	−27.96 ± 9.35^∗^‡	−2.00 ± 3.77
*Venous O_2_ Saturation (%)*		*% change from baseline*						
VEH	65.35 ± 11.72	4.17 ± 15.6	1.92 ± 16.26	−69.41 ± 16.87‡	−69.83 ± 15.91‡	−70.97 ± 16.73‡	−56.61 ± 25.82‡	−0.13 ± 1.63
5-HMF-20	70.26 ± 5.8	−3.40 ± 6.91	−0.10 ± 6.17	−34.78 ± 12.76^∗^‡	−43.51 ± 25.17‡	−38.05 ± 26.35‡	−38.41 ± 27.76‡	5.29 ± 12.22
5-HMF-40	68.24 ± 7.22	−2.65 ± 8.55	−6.36 ± 8.51	−44.81 ± 11.35‡	−63.69 ± 23.23‡	−52.09 ± 22.76‡	−61.31 ± 29.39‡	5.13 ± 5.61

*Groups: VEH (100 ml NS), 5-HMF-20 (20 mg/kg 5-HMF dissolved in 100 ml NS) and 5-HMF-40 (40 mg/kg 5-HMF dissolved in 100 ml NS). FiO_2_, FiO_2_, Fraction of Inspired Oxygen. Baseline absolute values in column with gray background and percentage change from baseline in remaining columns ± SD. ^∗^ vs. VEH (same time point; p ≤ 0.05); ^#^ vs. VEH (same time point; p ≤ 0.01); ^‡^ vs. baseline (p ≤ 0.05).*

### 5-HMF Attenuates Deleterious Effects of Hypoxia on Cardiopulmonary Outcomes and Blood Oxygenation

All values are normalized to baseline, the graphical representation of which are displayed in Figure 3 and tabulated in Table 2. Evident are significantly increased HR, CO, and PAP as well as decreased SaO_2_ and SvO_2_ amongst all groups during NH when compared to baseline values.

**FIGURE 4:**
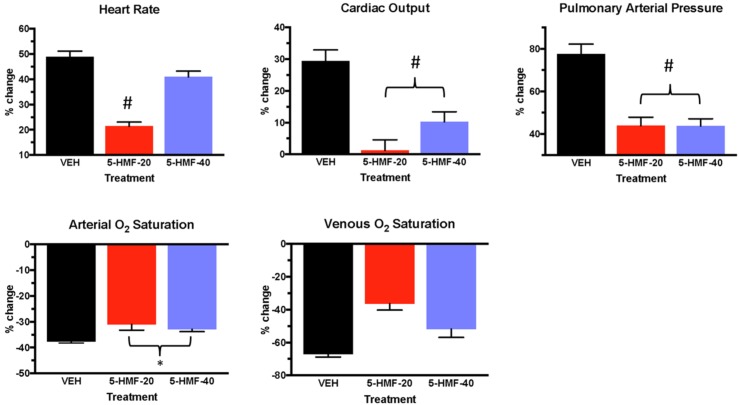
Cardiopulmonary outcomes during the first 30 min of hypoxia exposure in swine. Groups: VEH (100 ml NS), 5-HMF-20 (20 mg/kg 5-HMF dissolved in 100 ml NS) and 5-HMF-40 (40 mg/kg 5-HMF dissolved in 100 ml NS). All values are mean %-change in parameter (vs. mean baseline value) during the first 30 min of hypoxia exposure ± SD. ^∗^ vs. VEH (*p* ≤ 0.05); ^#^ vs. VEH (*p* ≤ 0.01).

In the first 30 min of NH (Figure 4 and Table 2), SaO_2_ was 39, 30, and 33% lower in VEH, 5-HMF-20, and 5-HMF-40 groups respectively, with significantly less decrement in 5-HMF-20 and 5-HMF-40 compared to VEH. SvO_2_ was 69, 34, and 44% lower in VEH, 5-HMF-20 and 5-HMF-40 groups, with significantly less decrement in the 5-HMF-20 group compared to VEH. HR increased by 47, 20, and 39% in VEH, 5-HMF-20 and 5-HMF-40 groups, with the 5HMF-20 being significantly less increased than VEH. CO increased by 26, 1, and 10% respectively in VEH, 5-HMF-20, and 5-HMF-40 groups; with 5-HMF-20 and 5-HMF-40 being significantly lower than VEH. PAP increased by 80, 45, and 40% in VEH, 5-HMF-20, and 5-HMF-40 groups respectively, with the 5-HMF-20 and 5-HMF-40 being significantly lower than VEH.

At the end of the 120 min hypoxia exposure, SaO_2_ was decreased by 40, 28, and 28% in VEH, 5-HMF-20, and 5-HMF-40 groups respectively, with significantly less decrement in both the 5-HMF-20 and 5-HMF-40 groups compared to VEH. SvO_2_, HR, and CO were not significantly different between groups. PAP was increased by 91, 43, and 49% in VEH, 5-HMF-20 and 5-HMF-40 groups respectively, with the 5-HMF-20 group being significantly lower than VEH.

### Arterial and Venous Blood Gas Outcomes, but Not Blood Chemistry, Are Affected by 5-HMF Treatment During Acute Normoxic and Hypoxic Exposure

Arterial and venous blood gas samples were analyzed before and after VEH/5-HMF treatment during normoxia and hypoxia (Table 3). Hypoxia resulted in significant reductions in arterial and venous pCO_2_, pO_2_ and SaO_2_ in all groups. There were no significant effects of 5-HMF on total white blood cell, hemoglobin, or platelets (data not shown) during normoxia or NH.

## Discussion

The purpose of this investigation was to determine the effects of 2 defined doses of 5-HMF on cardiopulmonary outcomes and P_50_ during an acute bout of severe hypoxia in a large animal model. We demonstrated that the administration of 5-HMF invoked a significant decrease in P_50_ and was associated with both a higher peripheral SaO_2_ and lower CO, HR, and PAP with acute NH. Additionally, 5-HMF administration was associated with a trend toward improved mortality during a NH challenge.

Upon exposure to NH, the anticipated decreased SaO_2_, increased HR, increased CO, elevated PAP, and reduced SvO_2_ was uniformly observed in all control animals. In the first 30 min of NH, 5-HMF was associated with higher SaO_2_, lower CO and lower PAP. Additionally, SvO_2_ values demonstrated a mitigated reduction (higher SvO_2_) with 5-HMF treatment but did not reach significance. These findings suggest that 5-HMF treatment animals experienced less physiologic derangements than control animals during NH. Taken together, it can be interpreted that metabolic needs were being met during NH in the presence of 5-HMF, and that a higher CO during NH was simply not required.

Though the physiologic benefits with 5-HMF treatment in the initial 30 min of NH were clear, the benefits in the following 90 min of the NH challenge were less certain. Supporting the concept of less physiologic derangement with 5-HMF treatment during NH is the (unexpected and problematic) mortality with 5/7 animals in the control arm succumbing to the hypoxic challenge. Although mortality may be the ultimate, but not ideal, marker for deranged physiology, the current study was unfortunately not sufficiently powered to answer that specific outcome. This unexpected mortality had the effect of reducing the utility of physiologic measurements in the latter 90 min of NH. Many parameters in the time period of 30–120 min of NH (spanning 125–210 min of the experiment) remain interesting (such as SaO_2_), but limited conclusions can be drawn due to inherent “survival bias” (Abdulmalik et al., 2005) and large standard deviations. Additionally, attention is drawn to the variability in p50 change with our two doses of 5-HMF. Though 5-HMF reliably binds to HgB in *in vitro* settings, its *in vivo* partitioning into the red blood cell is not as well known (Lucas et al., 2011) and may lead to less reliable p50 (Jensen et al., 2007). Newer aromatic aldehydes, such as GBT 440 appear to have increased red cell partitioning, more reliable change in p50 and longer half-lives (Dufu et al., 2017).

Nonetheless, our results in this large animal (swine) model are congruent with data demonstrating benefits of 5-HMF during hypoxia in small animal models. In a mouse model of extreme altitude exposure (9500M), 5-HMF pretreatment improved mortality and blood brain barrier permeability as well as attenuated nerve cell deterioration in the hippocampus (Oksenburg et al., 2016) and stabilizes HIF-1 alpha during HH (Li et al., 2011). In a hamster model, 5-HMF applied to NH was associated with improved SaO_2_, CO, tissue PO_2_, and microvascular blood flow (Scott, 2011). This investigation also demonstrated that mice exposed to 5-HMF and NH had less hypoxic tissue staining in brain and heart using pimonidazole. In addition to the decreased P_50_ that 5-HMF effects, evidence exists for antioxidant effects, anti-inflammatory and antiproliferative properties (Kim et al., 2011; He et al., 2014). As such, 5-HMF has revealed benefits in patients of lung cancer surgery (Zhao et al., 2013), cerebral ischemia (Matzi et al., 2007), and exercise induced oxidation (Ya et al., 2017). Interestingly, in human exercise during NH when used as an antioxidant, 5-HMF combined with alpha ketoglutaric acid partially ameliorated exercise decrements (Klarod et al., 2015).

Interestingly, the increase in PAP during NH was attenuated by 5-HMF at both doses. Hypoxic pulmonary vasoconstriction (HPV) is a highly conserved response to alveolar hypoxia, matching ventilation to perfusion and optimizing gas exchange (Mariacher et al., 2014). This response is beneficial in reducing localized intrapulmonary shunt from conditions such as acute respiratory distress syndrome (ARDS) and pneumonia (Sommer et al., 2016), but whether it is advantageous in NH and HH has yet to be confirmed.

Hypoxic pulmonary vasoconstriction is achieved by vasoconstriction of the pre-capillary pulmonary vessels at the entrance to the acinus and to a lesser degree by constriction of the pulmonary venules in response to alveolar hypoxia and decreased pulmonary artery O_2_ tension (Mariacher et al., 2014). Within seconds of exposure to a hypoxic gas mixture there is an initial constrictor response followed by a more sustained phase lasting 30–180 min, and then again followed by a third phase of more pronounced pulmonary vasoconstriction (Mariacher et al., 2014). In our experiment, all experimental swine exhibited an initial increase in PAP and a more sustained elevation within the time courses previously reported in the literature. However, all animals treated with 5-HMF exhibited much less initial elevation than control animals and a suggestion of sustained improvement throughout the course of the NH challenge.

The effector cell of HPV appears to be the pulmonary artery smooth muscle cell (PASMC), but the sensing mechanism is less certain. Marshall and Marshall (1983) used an anterograde and retrograde perfused rodent lung to demonstrate that pre-capillary O_2_ tension increased vasoconstriction in the presence of alveolar hypoxia, hypothesizing that the sensing apparatus also resides within the PASMC (Sommer et al., 2016). In our work, the lower mPAP during the first 30 min of NH was associated with higher SvO_2_, but not a similar increase in partial pressure of O_2_ in the mixed venous blood. The current study suggests that the oxygen content in the pulmonary precapillary system is an important driver of pulmonary vasoconstriction, and not simply pO_2_.

**Table 3:** Effect of 5-HMF on arterial and venous blood gas outcomes during normoxia and hypoxia exposures in swine.

Treatment	Arterial Blood Gas					Venous Blood Gas				
	Time (FiO_2_)									
	30 (0.21)	90 (0.21)	150 (0.10)	210 (0.10)	240 (0.21)	30 (0.21)	90 (0.21)	150 (0.10)	210 (0.10)	240 (0.21)
	*pH*	*% change from baseline*				*pH*	*% change from baseline*			
VEH	7.45 ± 0.02	−0.35 ± 0.32	−1.05 ± 1.28	−1.43 ± 0.71	−1.06 ± 1.15	7.42 ± 0.02	−0.44 ± 0.47	−0.56 ± 0.69	−1.15 ± 0.78	−0.11 ± 0.00
5-HMF-20	7.42 ± 0.04	0.10 ± 0.30^∗^	−0.18 ± 0.74	−0.98 ± 1.40	−1.36 ± 1.24^‡^	7.38 ± 0.04	−0.15 ± 0.37	−0.98 ± 1.74	−0.92 ± 1.90	−1.23 ± 1.74
5-HMF-40	7.44 ± 0.04	−0.01 ± 0.45	−0.92 ± 1.06	−1.12 ± 1.32	−2.29 ± 1.07^‡^	7.4 ± 0.03	−0.13 ± 0.11	−0.81 ± 0.91	−1.29 ± 0.77^‡^	−1.79 ± 0.15^‡^
	*pCO_2_ (mmHg)*	*% change from baseline*				*pCO_2_ (mmHg)*	*% change from baseline*			
VEH	43.53 ± 3.56	−0.96 ± 6.54	−39.49 ± 8.99^‡^	−40.16 ± 11.51^‡^	−36.53 ± 3.94^‡^	48.6 ± 3.95	3.31 ± 12.50	−30.05 ± 9.72^‡^	−30.25 ± 4.50	−33.77 ± 0.00^‡^
5-HMF-20	43.76 ± 3.31	−5.13 ± 6.01	−33.6 ± 6.44^‡^	−43.55 ± 7.53^‡^	−38.65 ± 8.65^‡^	52.05 ± 1.93	−3.72 ± 3.49	−34.99 ± 2.77^‡^	−43.05 ± 4.77^∗‡^	−40.73 ± 10.76^‡^
5-HMF-40	43.8 ± 2.66	−7.58 ± 5.47	−35.31 ± 6.85^‡^	−36.84 ± 6.32^‡^	−34.52 ± 16.15^‡^	49.8 ± 2.86	−4.82 ± 5.81	−29.24 ± 8.38^‡^	−39.23 ± 4.86^‡^	−47.92 ± 24.87^‡^
	*pO_2_ (mmHg)*	*% change from baseline*				*pO_2_ (mmHg)*	*% change from baseline*			
VEH	85.78 ± 10.34	−8.58 ± 7.82	−69.03 ± 2.8^‡^	−64.69 ± 2.30^‡^	−26.02 ± 35.03	41.8 ± 3.37	−7.66 ± 7.39	−53.6 ± 21.28^‡^	−54.41 ± 7.99^‡^	−29.36 ± 0.00
5-HMF-20	83.53 ± 10.46	−1.92 ± 13.91	−65.49 ± 3.79^‡^	−59.14 ± 9.04^‡^	7.64 ± 10.66^∗^	41 ± 3.62	−13.29 ± 5.39	−56.63 ± 7.64^‡^	−51.85 ± 11.91^‡^	−5.96 ± 22.70
5-HMF-40	95.9 ± 25.95	−13.11 ± 14.53	−69.85 ± 7.28^‡^	−72.88 ± 5.91^&‡^	−20.99 ± 15.39^&^	40.07 ± 4.23	−7.48 ± 5.57	−62.45 ± 5.99^‡^	−58.71 ± 6.20^‡^	24.89 ± 7.74
	*SO_2_ (mmHg)*	*% change from baseline*				*SO_2_ (mmHg)*	*% change from baseline*			
VEH	96.17 ± 3.62	−2.68 ± 4.44	−59.6 ± 8.57^‡^	−51.33 ± 3.12^‡^	−13.15 ± 21.89	67.4 ± 4.33	−11.78 ± 9.40	−68.94 ± 18.86^‡^	−74.09 ± 6.51^‡^	−39.86 ± 0.00
5-HMF-20	95.37 ± 2.21	−0.43 ± 3.18	−52.92 ± 8.61^‡^	−49.87 ± 14.28^‡^	0.45 ± 2.21	64.42 ± 6.49	−14.99 ± 6.71	−73.53 ± 4.21^‡^	−70.91 ± 7.84^‡^	−2.71 ± 16.62
5-HMF-40	96.23 ± 1.8	−1.36 ± 1.57	−61.04 ± 5.87^‡^	−64.7 ± 4.60^‡^	−6.32 ± 7.17	63.34 ± 5.77	−11.02 ± 6.83	−80.32 ± 2.71^‡^	−78.16 ± 6.17^‡^	10.17 ± 7.42^‡^

*Groups: VEH (100 ml NS), 5-HMF-20 (20 mg/kg 5HMF dissolved in 100 ml NS) and 5-HMF-40 (40 mg/kg 5HMF dissolved in 100 ml NS). FiO_2_, FiO_2_, Fraction of Inspired Oxygen. Baseline absolute values in column with gray background and percentage change from baseline in remaining columns ± SD. ^∗^ vs. VEH (p ≤ 0.05); ^#^ vs. VEH (p ≤ 0.01); ^&^ vs. 20 mg/kg 5-HMF (p ≤ 0.05); ^‡^ vs. baseline (p ≤ 0.05).*

Invoked mortality with an acute exposure to an FiO_2_ of 0.10 was unanticipated during this study and presents a significant limitation. An FiO_2_ of 0.10 was chosen for this investigation based on a swine model that utilized a 48 h exposure to an FiO_2_ of 0.10 that produced high altitude pulmonary edema but no lethality (Kleinsasser et al., 2003). However, in that work no invasive instrumentation was performed, and the animals were neither sedated nor restrained. These differences likely impacted our animals’ ability to physiologically compensate to NH (such as increased ventilation). In the current study, animals appeared to succumb to progressive right heart failure during the hypoxic challenge. This observation further supports the role of 5-HMF in mitigating pulmonary vasoconstriction, as those animals that succumbed to NH were not further studied. Whether a non-restrained and non-sedated animal would have realized similar benefits is not known and deserves further study. Additional limitations include the short NH exposure time, as the impact of 5-HMF outside of our time period studied would be useful. Furthermore, other mechanisms outside of changing P_50_ (such as anti-oxidative properties) were not explored further, but current investigations now underway in the lab are examining such possibilities.

Despite limitations in this work, the physiologic benefits of increasing O-H affinity with 5-HMF during acute NH coupled with a decrease in mortality (although non-significant) suggest a potential use of 5-HMF with NH. These findings combined with 5-HMF antioxidant benefits in humans in HH (Zhao et al., 2013) and evidence of cerebral protection in rodents during HH (Abdulmalik et al., 2005; Oksenburg et al., 2016), strongly suggest a role of 5-HMF in combating the derangements associated with severe HH.

## Conclusion

Our study determined that 5-HMF treatment increased O-H affinity, SaO_2_, and mitigated increases in PAP in this swine model of hypoxia. Further investigations into the effects of 5-HMF treatment during NH and HH are necessary and should include longer duration of therapy and exposure.

## Ethics Statement

The study protocol was reviewed and approved by the Walter Reed Army Institute of Research/Naval Medical Research Center Institutional Animal Care and Use Committee in compliance with all applicable Federal regulations governing the protection of animals in research.

## Author Contributions

RM and JS were responsible for the conception and design of the experiments. RM, NR, and JS were responsible for the completion of the experiments, data analysis, and interpretation of the results. RM, GC, and JS completed all figure preparation, statistical analyses, and draft of the manuscript. RM, GC, NR, and JS contributed to editing and revisions of the manuscript and approval of the final version.

## Disclaimer

JS and GC are currently military service members. This work was prepared as part of their official duties. Title 17 U.S.C. §105 provides that ‘Copyright protection under this title is not available for any work of the United States Government.’ Title 17 U.S.C. §101 defines a U.S. Government work as a work prepared by a military service member or employee of the U.S.Government as part of that person’s official duties.

## Conflict of Interest Statement

The authors declare that the research was conducted in the absence of any commercial or financial relationships that could be construed as a potential conflict of interest.
